# Mapping DWI signal reversal and long-term tissue outcomes following endovascular therapy in acute ischemic stroke

**DOI:** 10.1007/s00330-025-11943-0

**Published:** 2025-09-01

**Authors:** Thor Håkon Skattør, Cecilie Mørck Offersen, Terje Nome, Kine Mari Bakke, Brian Anthony B. Enriquez, Jonathan Frederik Carlsen, Mona Kristiansen Beyer, Atle Bjørnerud, Anne Hege Aamodt

**Affiliations:** 1 Department of Neurology, Oslo University Hospital, Oslo, Norway; 2 Institute of Clinical Medicine, University of Oslo, Oslo, Norway; 3 Division of Radiology and Nuclear Medicine, Oslo University Hospital, Oslo, Norway; 4 Department of Radiology, Copenhagen University Hospital, Rigshospitalet, Copenhagen, Denmark; 5 Department of Clinical Medicine, University of Copenhagen, Copenhagen, Denmark; 6 Department of Physics and Computational Radiology, Oslo University Hospital, Oslo, Norway; 7 Center for Lifespan Changes in Brain and Cognition, Department of Psychology, University of Oslo, Oslo, Norway; 8 Department of Neuroscience and Movement Science, The Norwegian University of Science and Technology, Trondheim, Norway

**Keywords:** Brain, Diffusion magnetic resonance imaging, Endovascular procedures, Ischemic stroke, Reperfusion

## Abstract

**Objectives:**

Diffusion-weighted imaging lesion reversal (DWI-R) is commonly observed on MRI after treatment for acute ischemic stroke. However, its extent across different brain regions post-endovascular therapy (EVT) and its long-term tissue-specific consequences are inadequately described in the literature. This study evaluated DWI-R across brain regions and tissue types and assessed long-term changes after 1 month.

**Materials and methods:**

This cohort study included acute ischemic stroke patients with MRI acquired before and 12 to 36 h after EVT. DWI lesions were segmented and co-registered to MNI space to generate probabilistic maps of DWI-R distribution. The probability of DWI-R was analyzed in relation to the involvement of white matter, cortical regions, and deep gray matter. Changes indicative of tissue damage were evaluated in a subgroup with follow-up imaging > 1 month post-EVT.

**Results:**

Of 565 consecutive EVT patients in the period January 2017-March 2022, 303 were included. DWI-R was observed in all major vascular territories. White matter showed 1.95 times higher probability of DWI-R compared to deep gray matter (*p* < 0.001), with no significant difference compared to cortical regions. Among 62 DWI-R cases with follow-up imaging, 29 (47%) showed no signal changes in areas of the initial lesion, with no significant difference between white and gray matter.

**Conclusions:**

Caution is advised when excluding patients from EVT based on restrictive diffusion, as both white and gray matter frequently responded to treatment in this study.

**Key Points:**

***Question*** *Understanding diffusion-weighted imaging reversal following endovascular therapy is crucial for stroke diagnostics, yet its regional distribution and long-term tissue-specific consequences remain poorly characterized*.

***Findings*** *Diffusion-weighted imaging reversal occurred throughout the brain, often persisted, and did not significantly differ between white matter and cortical regions*.

***Clinical relevance*** *Restrictive diffusion on MRI should be used with caution to exclude acute ischemic stroke patients from endovascular therapy, as such changes often respond to treatment both in white and gray matter*.

**Graphical Abstract:**

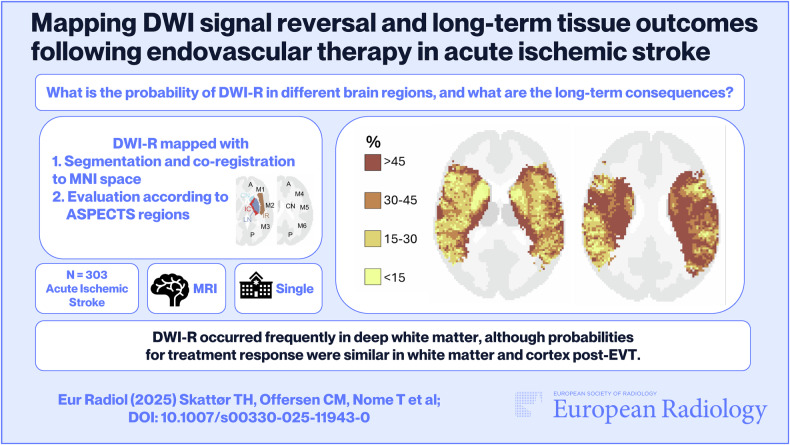

## Introduction

Restrictive diffusion observed on MRI in diffusion-weighted imaging (DWI) sequences has been used as a marker of the infarct core in acute ischemic stroke patients [1, 2]. However, in patients who undergo successful reperfusion therapy, DWI lesion reversal (DWI-R) is commonly observed. DWI-R refers to the resolution of previously seen restrictive diffusion on MRI following treatment, suggesting potential tissue recovery [3–6]. As gray matter (GM) has a higher metabolic rate and is generally more susceptible to ischemic injury than white matter (WM), the probability of DWI-R might be different in these tissues [7, 8]. In previous studies, infarcts involving WM were associated with worse outcomes than those confined to GM [9, 10]. Furthermore, previous publications have suggested that DWI-R could be primarily a transient phenomenon [1, 11–14]. Only sparse knowledge exists on the fate of DWI-R lesions after the first few days post-endovascular therapy (EVT), and even less information exists on alterations in late damage across different tissue types. A detailed understanding of the distribution of DWI-R and its long-term consequences is crucial for improving the accuracy of stroke diagnostics. This knowledge can help ensure appropriate patient selection for EVT, minimize the risk of excluding patients who could benefit, and improve overall treatment outcomes.

This study provides a novel perspective on DWI-R by systematically mapping its spatial distribution using standardized brain maps that illustrate its probability across various regions. Our primary aim was to examine the distribution of DWI-R in WM, GM, and across different anatomical locations in acute ischemic stroke patients undergoing EVT. Our secondary aim was to assess the long-term fate of these DWI-R lesions, investigating whether tissue type influences subsequent damage. By integrating spatial mapping with long-term imaging analysis, our approach offers new insights into the tissue-specific dynamics of DWI-R and its implications for EVT decision-making.

## Materials and methods

### Study design and population

The present study received approval from the regional ethics committee (REK 2015/1844). Written consent was obtained from either the patient or their legally authorized representative. This retrospective substudy is part of an ongoing cohort study of consecutive patients undergoing EVT at a tertiary center (NCT06220981). [15–17]. Inclusion criteria include age 18 years or older, patients with ischemic stroke and cerebral arterial occlusion treated with endovascular reperfusion regardless of stroke severities and vascular distributions. For this substudy, we included patients who underwent MRI scans with DWI sequences to guide EVT decisions and who were rescanned 12–36 h after EVT between January 2017 and March 2022. Only patients with DWI scans of insufficient quality were excluded. MRI and NCCT scans from > 1 month post-stroke were examined when available. Clinical parameters, including National Institutes of Health Stroke Scale (NIHSS) score on admission, modified Rankin Scale (mRS) score at baseline and 90-day follow-up were registered prospectively.

### Imaging analyses

DWI scans from before and after EVT were analyzed for DWI-R with two distinct methods.

**Fig. 1:**
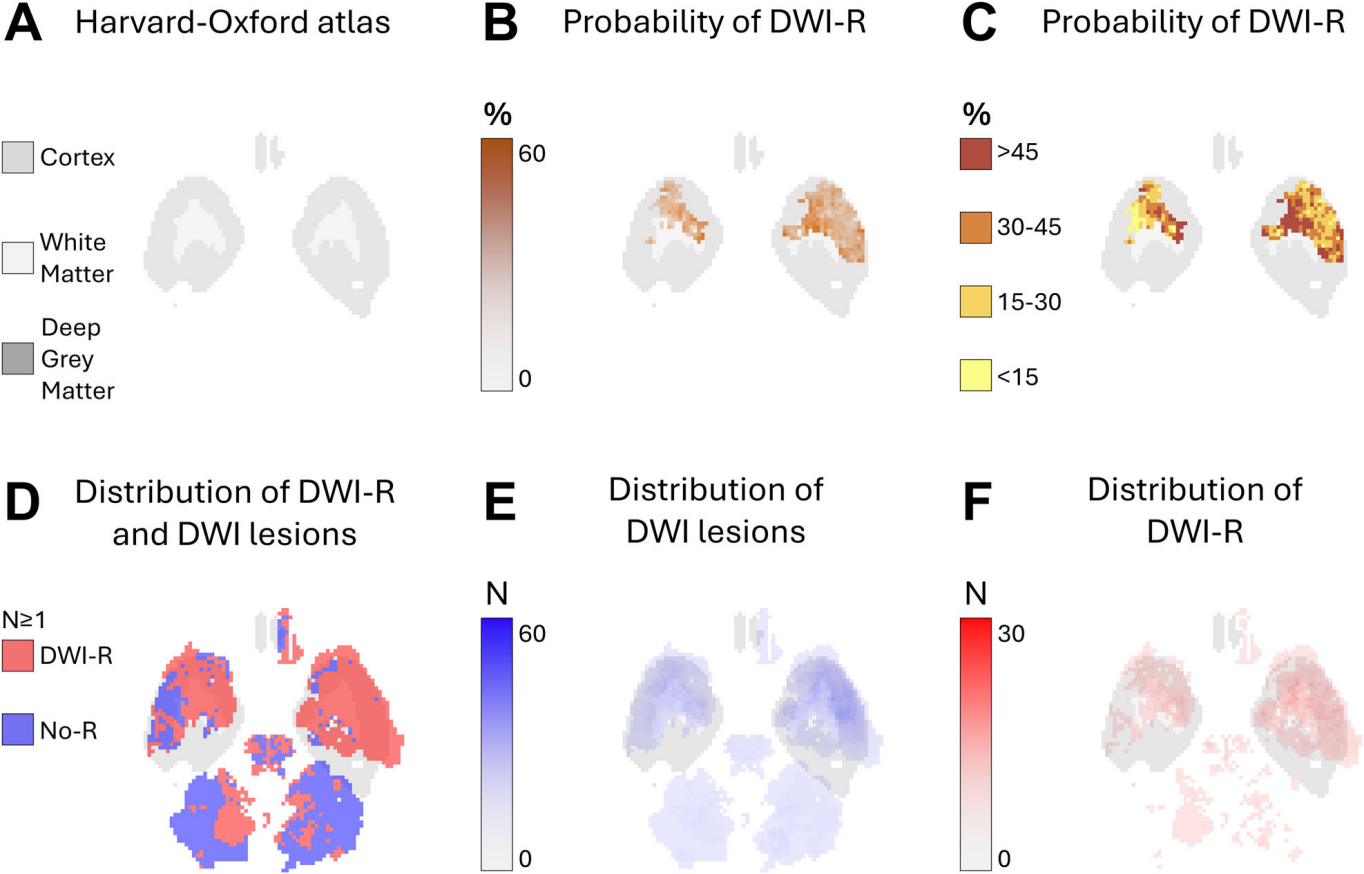
Axial probabilistic maps at the level of the temporal poles and cerebellum in MNI space. **A** Tissue regions extracted from the HarvardOxford-sub-maxprob-thr25-2mm atlas, derived from the Harvard-Oxford cortical and subcortical structural probabilistic atlases (also used as the background for **B**–**F**). **B**, **C** Graphical representation of the probability of diffusion-weighted imaging lesion reversal (DWI-R). Higher probabilities are shown in darker colors. Only voxels that were part of a DWI lesion in at least five patients on MRI performed prior to endovascular therapy (EVT) are included. **D** Spatial distribution of DWI lesions (blue) and DWI-R lesions (red), showing all voxels affected in at least one patient. **E** Number of patients with a DWI lesion per voxel on pre-EVT MRIs. **F** Number of patients with DWI-R per voxel

**Fig. 2:**
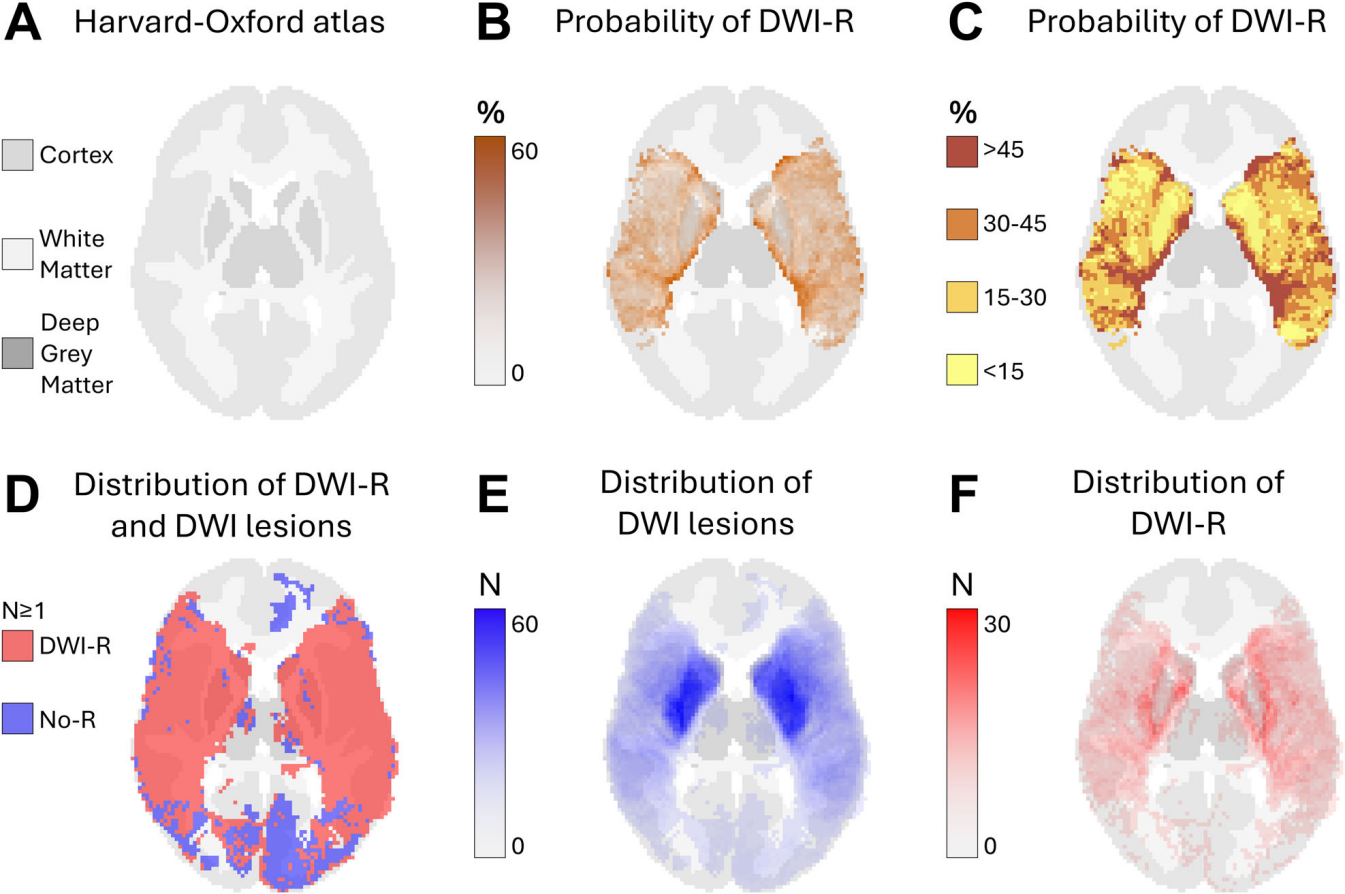
Axial probabilistic maps at the level of the lower basal ganglia in MNI space. **A** Tissue regions extracted from the HarvardOxford-sub-maxprob-thr25-2mm atlas, derived from the Harvard-Oxford cortical and subcortical structural probabilistic atlases (also used as the background for **B**–**F**). **B**, **C** Graphical representation of the probability of diffusion-weighted imaging lesion reversal (DWI-R). Higher probabilities are shown in darker colors. Only voxels that were part of a DWI lesion in at least five patients on MRI performed prior to endovascular therapy (EVT) are included. **D** Spatial distribution of DWI lesions (blue) and DWI-R lesions (red), showing all voxels affected in at least one patient. **E** Number of patients with a DWI lesion per voxel on pre-EVT MRIs. **F** Number of patients with DWI-R per voxel

**Fig. 3:**
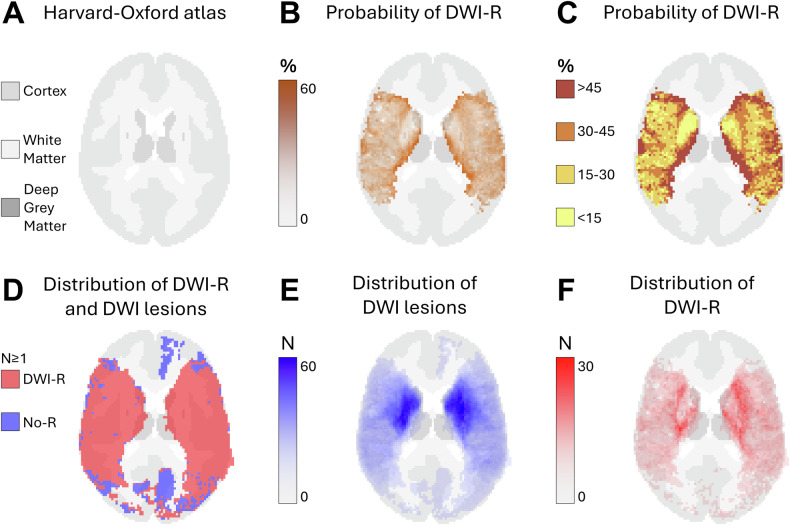
Axial probabilistic maps at the level of the upper basal ganglia in MNI space. **A** Tissue regions extracted from the HarvardOxford-sub-maxprob-thr25-2mm atlas, derived from the Harvard-Oxford cortical and subcortical structural probabilistic atlases (also used as the background for **B**–**F**). **B**, **C** Graphical representation of the probability of diffusion-weighted imaging lesion reversal (DWI-R). Higher probabilities are shown in darker colors. Only voxels that were part of a DWI lesion in at least five patients on MRI performed prior to endovascular therapy (EVT) are included. **D** Spatial distribution of DWI lesions (blue) and DWI-R lesions (red), showing all voxels affected in at least one patient. **E** Number of patients with a DWI lesion per voxel on pre-EVT MRIs. **F** Number of patients with DWI-R per voxel

**Fig. 4:**
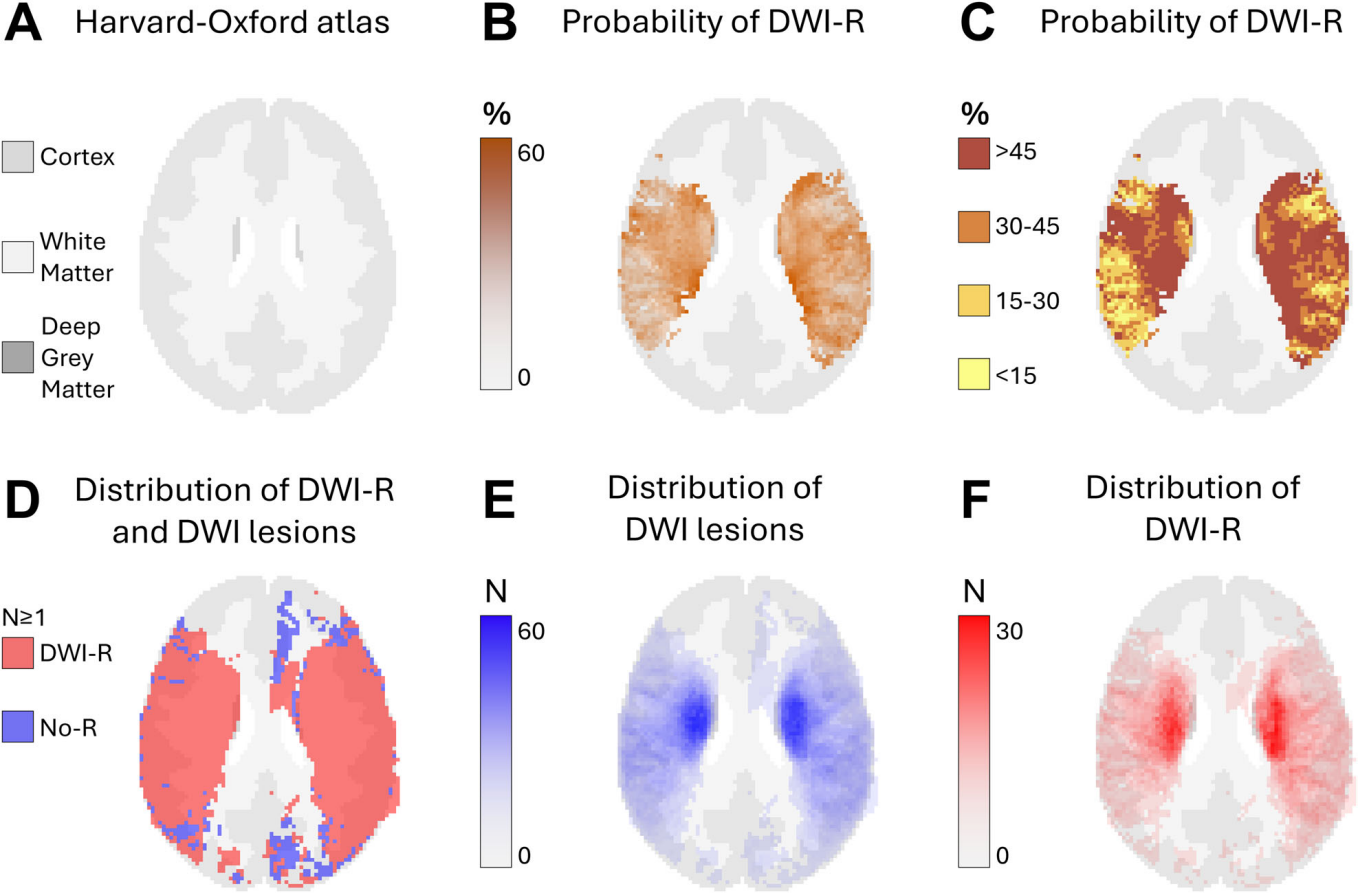
Axial probabilistic maps at the level of the inferior centrum semiovale in MNI space. **A** Tissue regions extracted from the HarvardOxford-sub-maxprob-thr25-2mm atlas, derived from the Harvard-Oxford cortical and subcortical structural probabilistic atlases (also used as the background for **B**–**F**). **B**, **C** Graphical representation of the probability of diffusion-weighted imaging lesion reversal (DWI-R). Higher probabilities are shown in darker colors. Only voxels that were part of a DWI lesion in at least five patients on MRI performed prior to endovascular therapy (EVT) are included. **D** Spatial distribution of DWI lesions (blue) and DWI-R lesions (red), showing all voxels affected in at least one patient. **E** Number of patients with a DWI lesion per voxel on pre-EVT MRIs. **F** Number of patients with DWI-R per voxel

**Fig. 5:**
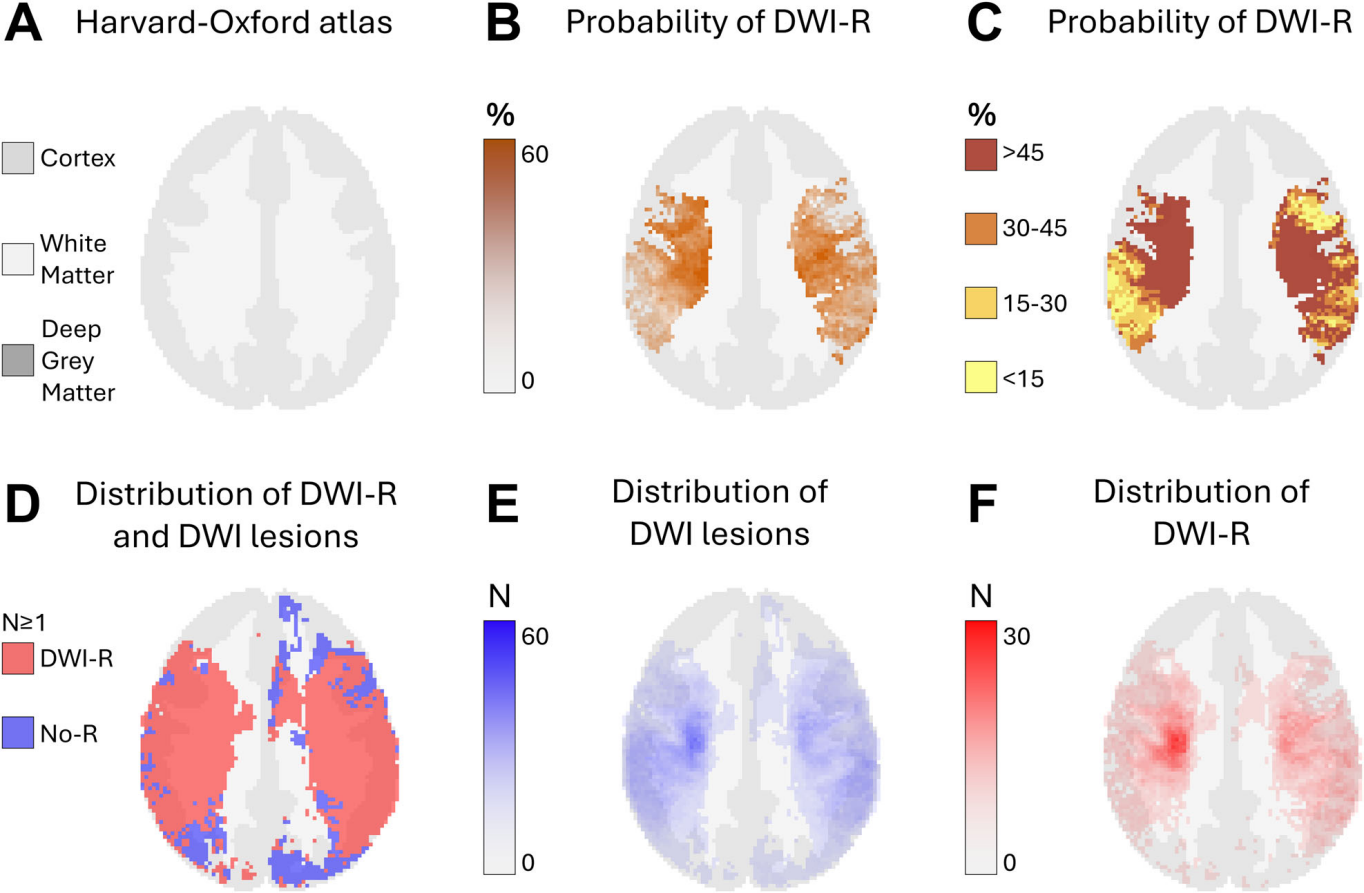
Axial probabilistic maps at the level of the superior centrum semiovale in MNI space. **A** Tissue regions extracted from the HarvardOxford-sub-maxprob-thr25-2mm atlas, derived from the Harvard-Oxford cortical and subcortical structural probabilistic atlases (also used as the background for **B**–**F**). **B**, **C** Graphical representation of the probability of diffusion-weighted imaging lesion reversal (DWI-R). Higher probabilities are shown in darker colors. Only voxels that were part of a DWI lesion in at least five patients on MRI performed prior to endovascular therapy (EVT) are included. **D** Spatial distribution of DWI lesions (blue) and DWI-R lesions (red), showing all voxels affected in at least one patient. **E** Number of patients with a DWI lesion per voxel on pre-EVT MRIs. **F** Number of patients with DWI-R per voxel

**Method 1**—**Segmentation:** In Method 1, we performed segmentation on high b-value maps (acquired b1000 and calculated b1500) using the nordicICE software with thresholding tools [18] to assess DWI-R. DWI lesions with an apparent diffusion coefficient (ADC) difference of ≥ 100 × 10^−6^ mm²/s from the contralateral side were included. DWI images and corresponding segmented lesion masks were co-registered to MNI space (ICBM152) with 2 mm isotropic voxels using the ANTsPy library [19–21]. MNI space provides a standardized reference framework for brain imaging data, enabling comparison across different subjects. From these co-registered masks, new segments were derived to differentiate DWI-R from parts of the DWI lesion that remained on images 12–36 h after EVT. For each voxel in MNI space, the proportion of patients with DWI lesions and DWI-R was calculated. The probability of DWI-R in WM, deep GM, and cortical regions was assessed by probabilistic voxel-based maps with intensities corresponding to DWI lesions and DWI-R (Figs. 1–5). The maps were applied to the HarvardOxford-sub-maxprob-thr25-2mm atlas, from Harvard-Oxford cortical and subcortical structural probabilistic atlases [22]. For voxels with initial DWI lesions in at least five patients, the probability of DWI-R was also mapped (Figs. 1–5). Independent segmentation was performed on a subset of 70 consecutive DWI studies by two experienced neuroradiologists (T.H.S., T.N.), who were blinded to clinical and procedural data. Inter-rater reliability, measured by the Dice coefficient, was high at 0.852 (95% CI 0.834–0.880). Discrepancies were resolved by consensus when the Dice coefficient was less than 0.7. Following consensus, one rater (THS) segmented the remaining 420 studies and the segments from this rater were used in the final analyses.

**Method 2**—**Evaluation:** In Method 2, we evaluated the locations of DWI-R through visual comparison of high b-value maps and ADC maps before and after EVT. DWI lesions and DWI-R locations were recorded in a database corresponding to DWI-Alberta stroke program early CT score (ASPECTS) areas [23–25] and to vascular territories supplied by the anterior cerebral artery, the posterior cerebral artery and infratentorial locations. We registered whether the DWI-R appeared in deep WM or in superficial regions, including cortical GM with or without superficial subcortical WM involvement. To mark an area as affected, the DWI lesion’s largest diameter needed to be at least 10 mm and have an ADC at least 100 × 10^−^⁶ mm²/s lower than the corresponding tissue on the contralateral side. Two experienced neuroradiologists (T.H.S., T.N.) conducted evaluations of a subset of 140 consecutive DWI scans, blinded to procedural and clinical outcomes. Inter-rater reliability was tested and found to be excellent, with 94.3% agreement on a linear-weighted kappa test on DWI-ASPECTS scores, kappa = 0.79 (95% CI 0.73–0.84). The remaining 466 images were assessed by one neuroradiologist (T.H.S.), and the results from this rater were used in further analysis. For cases where DWI-R was identified, a third radiologist (C.M.O.), who was blinded to the previous registrations, conducted a re-analysis of the images. Any discordance between the raters was resolved by consensus before final analysis.

**Cross-check:** As a quality assurance, all examinations identified as having DWI volume > 10 mL with Method 1 but not identified as DWI-R with Method 2 were reassessed. All these cases were found to represent the sum of multiple areas, each below the threshold defined in Method 2 (< 10 mm). Consequently, no new lesions were added to the database of Method 2.

**Follow-up images:** Follow-up images acquired > 1 month post-EVT were independently examined by two raters (THS, CMO) for DWI-R lesions identified with Method 2. Routine follow-up imaging at 3 months post-EVT was conducted as part of the main study for patients treated between 2017 and 2018. Additionally, follow-up imaging performed outside this time frame was included when available. The region with DWI-R was assessed on FLAIR and/or T2 sequences when MRI were available and on non-contrast computed tomography (NCCT) if this was the only available modality. The area of the initial DWI lesion was compared to the corresponding area on the contralateral side of the brain using 3 categories; 0 if no signal changes in MRI, attenuation changes on NCCT, or parenchymal volume loss; 1 if < 50% signal- or attenuation changes of the original lesion area, and 2 if > 50% signal- or attenuation changes of the original lesion area, or signs of atrophy. To evaluate whether ADC differed in DWI-R lesions with varying outcomes on follow-up, ADC was measured on the initial MRI. The measurement was taken from a centrally located region of DWI reversal, as visually identified by the rater, representing the lower end of the ADC distribution rather than the lesion’s mean ADC. To account for scanner and sequence variations, ADC differences were calculated relative to the corresponding contralateral tissue. All measurements were performed using the integrated tool in Sectra PACS.

### Statistical analysis

Statistical analyses were performed using SciPy v.1.11.4 and SPSS Statistics v.29. When comparing differences in continuous variables, the distribution was assessed for normality, and a Mann–Whitney U test or Student’s T-test was selected with respect to their distribution. Categorical data were evaluated with a Chi-Square test. Ordinal categorical data were tested with a Mann–Whitney U test.

## Results

During the inclusion period, 565 patients undergoing EVT were registered in the main study. Among them, 314 had available MRI examinations. However, 11 patients were excluded due to insufficient DWI quality caused by motion artifacts, leaving 303 patients for inclusion in this study. Of these, 245 patients (80.9%) had MRI data available for assessments in Method 1, while data were unavailable for the remaining patients due to technical issues with segmentation and co-registration processes. Demographic, clinical and radiological characteristics of included patients are shown in Table 1. A comparison of participants included in Method 1 and those not, revealed no significant differences in variables, except that MRI examinations from local hospitals were more frequently of insufficient quality for segmentation and co-registration (supplementary material Table S1).

**Table 1:** Background characteristics

Demographics, clinical and imaging characteristics	*N* = 303
Mean age, years (SD)	68.9 (12.6)
Male (%)	165 (54.5)
Anterior circulation occlusion	
Large vessel (%)	195 (64.4)
Internal carotid artery (%)	72 (23.8)
Medial cerebral artery, M1 segment (%)	122 (40.3)
Medium vessel (%)	77 (25.4)
Medial cerebral artery, M2 segment (%)	63 (20.8)
Medial cerebral artery, M3 segment (%)	13 (4.3)
Anterior cerebral artery (%)	1 (0.3)
Posterior circulation occlusion (%)	31 (10.2)
Basilar artery (%)	27 (8.9)
Posterior cerebral artery (%)	4 (1.3)
Intravenous thrombolysis (%)	166 (55.0)
Median NIHSS on admission (IQR)	12 (7–17)
Median time ictus to MRI pre-EVT, minutes (IQR)	224 (175–312)
Median time MRI pre to recanalization, minutes (IQR)	95 (75–137)
Median time ictus to recanalization, minutes (IQR)	335.5 (274.75–437.5)
Median time recanalization to MRI post, hours (IQR)	21.6 (18.5–25.0)
Median time to follow-up MRI/NCCT, days (IQR)	102 (90–115)
Median DWI-ASPECTS MRI pre-EVT (IQR)	7 (6–8)
Median DWI-ASPECTS MRI post-EVT (IQR)	7 (5–8)
mTICI ≥ 2b (%)	283 (93.4)
mTICI ≥ 2c (%)	205 (67.7)
Heidelberg classification score post-EVT	
1a&1b petechial (%)	101 (33.3)
1c parenchymal small (%)	39 (12.9)
2 parenchymal large (%)	19 (6.3)
3c subarachnoid (%)	28 (9.2)
Pre-stroke mRS ≤ 2 (%)	292 (96.4)
3-month mRS ≤ 2 (%)	210 (70.9)
Pre-EVT imaging	
MRI scanner at local hospital (%)	22 (7.3)
1.5-Tesla MRI scanner at intervention center (%)	270 (89.1)
3-Tesla MRI scanner at intervention center (%)	11 (3.6)
Post-EVT imaging at intervention center	
1.5-Tesla MRI (%)	295 (97.4)
3-Tesla MRI (%)	8 (2.6)

Background characteristics and imaging features of the study cohort

### Locations and probability of DWI-R

**Fig. 6:**
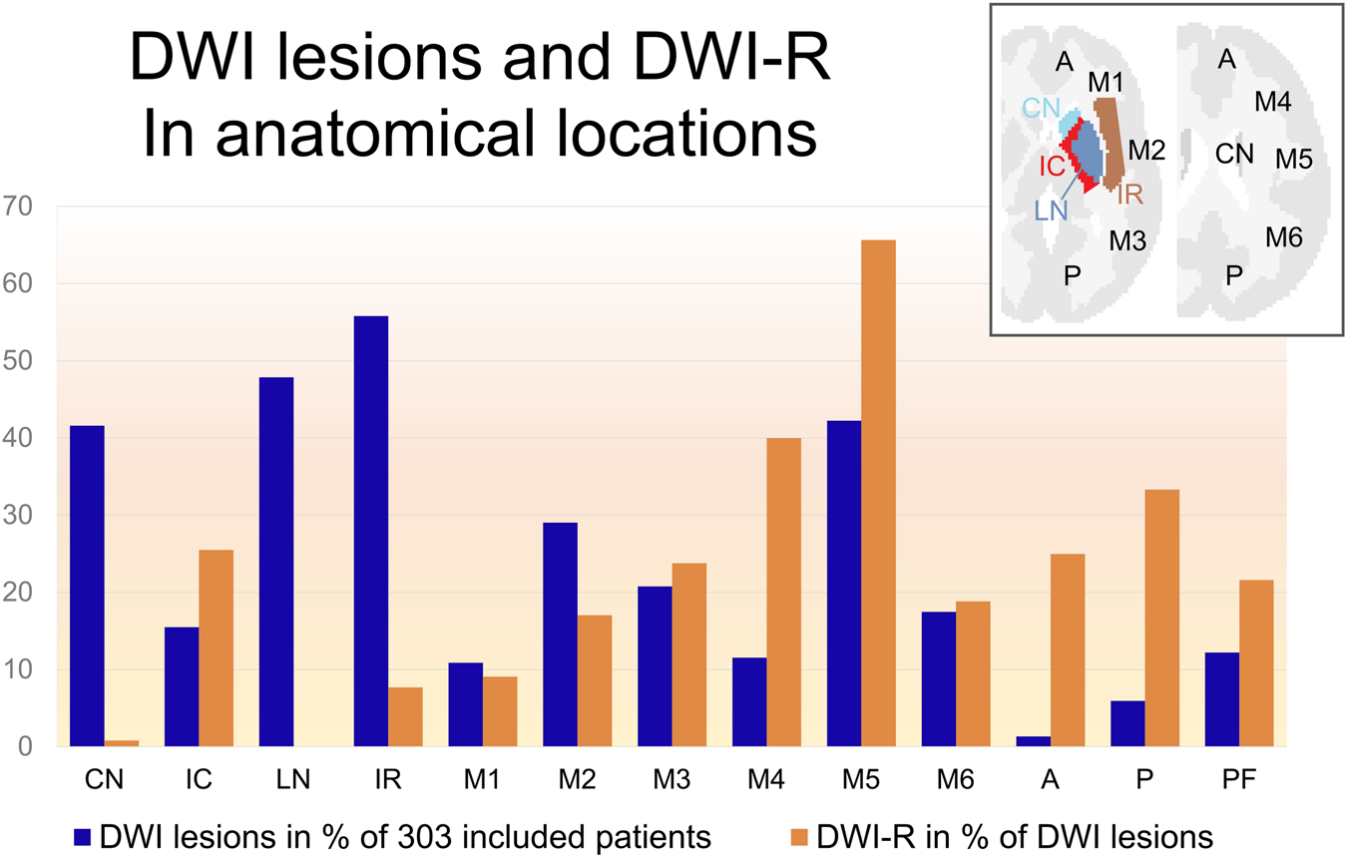
Rates of DWI lesions on MRI performed prior to endovascular therapy and the rate of diffusion-weighted imaging lesion reversal (DWI-R) found in Method 2. Data are analyzed across each Alberta Stroke Program Early CT Score (ASPECTS) region, as well as in areas corresponding to the vascular territories of the anterior cerebral artery, posterior cerebral artery, and posterior fossa. CN, caudate nucleus; IC, internal capsule; LN, lentiform nucleus; IR, insular ribbon; M1-M6, vascular territory of middle cerebral artery corresponding to ASPECTS region M1-M6; A, vascular territory of anterior cerebral artery; P, vascular territory of posterior cerebral artery; PF, posterior fossa; DWI, diffusion-weighted imaging; DWI-R, diffusion-weighted imaging lesion reversal

We observed DWI-R across all major cerebral vascular territories, as shown in the mapped distribution in Figs. 1–5 and the electronic supplementary material. DWI-R was absent only in regions with none or very few baseline DWI lesions. The figures also illustrate the probability of DWI-R mostly confined to middle cerebral artery territories with sufficient baseline lesions (≥ 5) for probability calculations. We found a high probability of DWI-R in deep WM, particularly in the internal capsule and its extensions through the corona radiata, whereas only minimal DWI-R was observed in the basal ganglia. The overall probability of DWI-R for voxels in our dataset was 41.5% in WM, 20.8% in deep GM and 30.9% in cortical regions (Table 2). When assessing probability of DWI-R in selective tissues across individual patients, we found 1.95 times higher median probability of DWI-R in WM voxels (35.8%) compared to deep GM (18.4%) (*p* < 0.001, Mann–Whitney U test), but no significant difference between WM and cortical regions (35.1%) (Table 2). Consistent with the findings on the probabilistic maps, the highest probability of DWI-R was found in ASPECTS regions 4 and 5 with Method 2 (Table 3 and Fig. 6). A total of 118 patients (38.9%) were found to have ≥ 10 mm DWI-R with this method whereof 76 (64.4%) had DWI-R in the internal capsule or corona radiata alone or in combination with other locations. Only one (0.8%) significant DWI-R lesion (≥ 10 mm) was observed in the ASPECTS regions corresponding to the caudate nucleus and the lentiform nucleus.

**Table 2:** Regional probability of DWI lesion reversal

	White matter	Cortical regions	Deep gray matter
Probability of DWI-R for all included voxels	41.5%	30.9%	20.8%
The median probability of DWI-R across individual patients (IQR)	35.8% (19.8–61.8)	35.1% (14.1–67.6)	18.4% (7.7–42.2)
Difference from white matter across individual patients	-	−2.0% (*p* = 0.77)	−48.6% (*p* < 0.001)

This table presents the probability of diffusion-weighted imaging lesion reversal (DWI-R) for voxels in white matter, deep gray matter, and cortical regions, as determined by Method 1. The first row indicates the overall probability of DWI-R for all included voxels in each brain region; as an aggregate measure, it may be influenced by outliers. The second row shows the median probability of DWI-R across individual patients, providing a measure that may better represent a typical patient. The third row presents the differences in probability relative to white matter, along with the corresponding significance levels. Significance was assessed using the Mann–Whitney U test

**Table 3:** Regional distribution of DWI lesion reversal

Region	DWI lesions pre-EVT *N* (% of all patients)	DWI-R lesions *N* (% of DWI lesions)	DWI-R involving white matter *N* (% of DWI-R lesions)	DWI-R involving gray matter *N* (% of DWI-R lesions)
Caudate nucleus	126 (41.6)	1 (0)	0 (0)	1 (100)
Internal capsule	47 (15.5)	12 (25.5)	12 (100)	0 (0)
Lentiform nucleus	145 (47.9)	0 (0)	-	-
Insular ribbon	169 (55.8)	13 (7.7)	0 (0)	13 (100)
M1- Vascular territory of middle cerebral artery	33 (10.9)	3 (9.1)	2 (66.7)	3 (100)
M2- Vascular territory of middle cerebral artery	88 (29.0)	15 (17.0)	4 (26.7)	15 (100)
M3- Vascular territory of middle cerebral artery	63 (20.8)	15 (23.8)	13 (86.7)	8 (53.2)
M4- Vascular territory of middle cerebral artery	35 (11.6)	14 (40.0)	11 (78.6)	7 (50.0)
M5- Vascular territory of middle cerebral artery	128 (42.2)	84 (65.6)	79 (94.1)	32 (38.1)
M6- Vascular territory of middle cerebral artery	53 (17.5)	10 (18.9)	10 (100)	6 (60.0)
Vascular territory of anterior cerebral artery	4 (1.3)	1 (25.0)	0 (0)	1 (100)
Vascular territory of posterior cerebral artery	18 (5.9)	6 (33.3)	1 (16.7)	5 (83.3.0)
Posterior fossa	37 (12.2)	8 (21.6)	4 (50.0)	4 (50.0)

This table presents the results from Method 2. The rate of MRI diffusion-weighted imaging (DWI) lesions prior to endovascular therapy (EVT) is shown in the first column, while the rate of diffusion-weighted imaging lesion reversal (DWI-R) is provided in the second column. The third and fourth columns present the rates of DWI-R involving white matter and gray matter, respectively. Data are analyzed across each Alberta Stroke Program Early CT Score (ASPECTS) region, as well as in areas corresponding to the vascular territories of the anterior cerebral artery, posterior cerebral artery, and posterior fossa

### Late damage in reversed lesions

Among the 118 (38.9%) patients with DWI-R lesions using Method 2, 55 (46.6%) patients had late follow-up MRI scans and an additional 7 (5.9%) patients had NCCT scans at median 102 (IQR 90–115) days after EVT (Table 4). A comparison of patients with and without follow-up images revealed no significant differences in the variables listed in Table 1, except for longer intervals from recanalization to post-EVT MRI in patients with follow-up imaging (supplementary material Table S1). We observed no signal or attenuation changes corresponding to the DWI-R lesions in 29 cases (46.8%), whereas minimal changes in < 50% of the initial lesion size were demonstrated in 21 cases (33.9%). Severe signal changes exceeding 50% of original DWI-R lesion or as evidence of atrophy were noted in 12 cases (19.4%). Although late-stage lesions were more common in WM, the difference between GM and WM was not statistically significant (Table 4). The ADC difference between the initial DWI lesion and the corresponding contralateral tissue was significantly greater in patients who developed severe changes on follow-up (> 1 month) compared to those without changes in the lesion location. However, this difference did not reach statistical significance in patients with minimal changes (Table 5). No significant differences in mRS were observed between patients with late damage compared to those without (Table 5).

**Table 4:** Late imaging outcomes in regions of DWI lesion reversal

	No lesion on follow-up, *N* (%)	< 50% of initial lesion, *N* (%)	> 50% of initial lesion, *N* (%)	*p*-value
All lesions	29 (46.8)	21 (33.9)	12 (19.4)	
Follow-up MRI	24 (43.6)	20 (36.4)	11 (20.0)	*p* = 0.36 (MRI vs NCCT)
Follow-up NCCT	5 (71.4)	1 (14.3)	1 (14.3)	
Lesions involving only GM	5 (83.3)	1 (16.7)	0 (0)	*p* = 0.072 (GM* vs WM*)
Lesions involving only WM	11 (34.4)	11 (34.4)	10 (31.3)	
Lesions involving GM	18 (60.0)	10 (33.3)	2 (6.7)	*p* = 0.15 (GM** vs WM**)
Lesions involving WM	24 (42.9)	20 (35.7)	12 (21.4)	

This table presents the number and rates of late signal changes observed in areas corresponding to diffusion-weighted imaging lesion reversal (DWI-R) on follow-up imaging conducted more than 1 month after endovascular therapy (EVT). The area of the initial diffusion-weighted imaging (DWI) lesion was compared to the contralateral side of the brain. No lesion on follow-up: Indicates the absence of signal changes on MRI, attenuation changes on non-contrast computed tomography (NCCT), or parenchymal volume loss. *<* 50% of initial lesion: Refers to cases where less than 50% of the area affected by the DWI-R lesion displayed signal or attenuation changes, with no evidence of atrophy. > 50% of initial lesion: Refers to cases where more than 50% of the area affected by the DWI-R lesion exhibited signal or attenuation changes, or there was evidence of atrophy. Differences in rates of late changes between NCCT and MRI, as well as between gray matter and white matter lesions, were tested using chi-square tests

*GM* gray matter, *WM* white matter; GM* lesions involving only gray matter, WM* lesions involving only white matter, GM** lesions involving gray matter, including those that affect both gray and white matter, WM** lesions involving white matter, including those that affect both gray and white matter

**Table 5:** ADC differences and clinical outcomes

	Mean ADC difference from corresponding tissue on contralateral side × 10^−^^6^ mm^2^/s (95% CI)	Difference in ADC compared to the group with no lesion on follow-up	*p*-value	Median mRS (IQR)	Difference in mRS compared to the group with no lesion on follow-up *p*-value
No lesion at follow-up, *n* = 29	−194.1 (−165.9, −225.3)	-	-	1 (1–2)	-
< 50% of initial lesion at follow-up, *n* = 21	−229.6 (−188.6, −273.7)	−35.5	0.32	1 (1–2)	1.0
≥ 50% of initial lesion at follow-up, *n* = 12	−260.6 (−207.0, −316.1)	−66.5	0.042	1 (0–2)	0.61

Comparison of patients with and without changes in the location of the initial diffusion-weighted imaging lesion reversal (DWI-R) on follow-up imaging > 1 month after endovascular therapy. The second column presents the difference in apparent diffusion coefficient (ADC) values between DWI-R lesions and the corresponding contralateral tissue from the initial MRI. The magnitude of this ADC difference was significantly higher in lesions that exhibited substantial late changes on follow-up imaging, as shown in the third column (difference from the group with no lesion on follow-up) and the fourth column (*p*-value). The fifth column displays modified Rankin Scale (mRS) scores at 3 months post-stroke, and the last column presents *p*-values from comparison against the group with no lesion on follow-up. Statistical comparisons were performed using the Mann–Whitney U test

## Discussion

In this study cohort of acute ischemic stroke patients treated with EVT, DWI-R was more extensive in WM than in deep GM, particularly abundant in the internal capsule and its extensions through the corona radiata, but sparse in the basal ganglia. The probability of DWI-R was comparable in WM and cortical regions, with a median probability of 36% for WM voxels and 35% for voxels in cortical regions. The follow-up images > 1 month after EVT showed no changes in areas of DWI-R lesions in approximately half of the cases and severe changes in less than 20%.

Infarcts that spare WM have been associated with better outcomes [9]. Accordingly, differentiated indications for EVT have been proposed for patients with infarcts involving WM compared to those confined to GM, particularly in the late time window [9, 10]. The probability for DWI-R was equally high in both cortical regions and WM in our study. This suggests that WM is likely to respond to treatment, and DWI from pre-treatment MRI should be used cautiously when distinguishing between the two types of final infarct distribution. We found that DWI-R was less likely in the deep GM. However, infarcts confined to the basal ganglia are often associated with relatively minor clinical deficits [26], suggesting that lesions in this area may have less significance in EVT triage. An explanation for the lower rate of DWI-R in deep GM could be the heightened vulnerability of the basal ganglia to ischemic stress, caused by their vascular supply from lenticulostriate end-arteries without collateral reserves [27]. The broad distribution of DWI-R observed in our study aligns with previous publications demonstrating that DWI-R occurs across various brain tissues [8, 28].

A study based on the DEFUSE 2 data [12] reported that DWI-R occurred in one-third of patients but was typically transient. However, persistent reversal was observed in more than half (53%) of the cases when assessing FLAIR signals in large lesions (> 10 mL) on MRI by day 5. Consistent with the findings of persistent reversal after a few days, our results showed no evidence of late signal changes in 29 of 62 cases (47%). Our findings support the notion that DWI signal is dynamic in the early hours following a stroke and aligns well with several previous publications showing improved clinical outcome in DWI-R patients after stroke treatment [2, 4, 29–31]. Furthermore, this underscores the recently documented benefits of treating large ischemic lesions, as demonstrated in several clinical trials [32–36]. Given the substantial amount of research highlighting the dynamic nature of DWI in the early hours following ictus, the argument that DWI lesions should be considered a reliable measure of the infarct core is highly problematic, particularly in the context of decisions regarding EVT [5, 31, 37]. We found significantly lower ADC values in lesions that exhibited severe changes on follow-up (12 of 62 cases, 19%), suggesting that ADC may help identify patients likely to benefit from treatment; however, further research in larger populations is needed to clarify its relationship with clinical outcomes [38]. No significant difference in mRS was observed, which may be due to the limited sample size.

Our results did not demonstrate a significant difference in late signal changes between WM and GM. However, this might be just a consequence of limited sample size. Another limitation was the different imaging modalities at the long-term follow-ups. Although NCCT is sufficient to detect severe infarct sequela, MRI is more sensitive to minor changes in brain tissue. However, the total number of follow-up where only NCCT was available is low, and the overall result for late damage is not significantly altered when only considering follow-up images with MRI, as shown in Table 4. The follow-up images were acquired based on clinical indications rather than being study-specific. However, basic characteristics of patients with and without follow-up were similar, except for a longer interval from recanalization to post-EVT MRI, plausibly coincidental. Thus, selection bias is probably minimal and likely overestimates late damage.

The processes of segmenting brain regions and registering individual brains to the atlas can introduce inaccuracies as no single brain perfectly matches the probabilistic atlases used in this study [39]. Additionally, probabilistic atlases may not fully represent our patient cohort in terms of age-related brain size and health status. Therefore, all DWI-R lesions of significant size were also visually evaluated by experienced neuroradiologists and categorized with respect to anatomical locations and tissue type. The consistency of findings across our methods strengthens our results.

The resolution of probabilistic atlases in MNI space is insufficient to distinguish between cortical GM and juxtacortical WM. However, since WM infarcts are associated with poorer outcomes, our key finding is that WM lesions also experience DWI-R, a conclusion supported confidently by both methods used in our study.

The frequencies and distribution of initial DWI lesions in our dataset align well with the vascular territories of vessels strongly supported by evidence for EVT, reflecting the characteristics of our study population. Provided maps with calculated probability of DWI-R were confined to the vascular territories of the middle cerebral arteries, as DWI lesions in other locations were not sufficiently prevalent in our dataset.

## Conclusion

In conclusion, DWI-R were observed throughout the brain following EVT in our cohort, with the most common locations in the deep WM, particularly within the internal capsules and their extensions into the corona radiata. Both WM and cortical DWI lesions frequently responded to EVT, with no significant difference in probability. These findings highlight the need for caution when excluding patients from EVT based on restrictive diffusion.

## Supplementary information

ELECTRONIC SUPPLEMENTARY MATERIAL

Video 1 Figure S1 (Axial)

Supplementary Information Figure S2 (Axial)

Supplementary Information Figure S3 (Axial)

Supplementary Information Figure S4 (Coronal)

Supplementary Information Figure S5 (Coronal)

Supplementary Information Figure S6 (Sagittal)

Supplementary Information Figure S7 (Sagittal)

## Data Availability

Data supporting the study findings are available from the corresponding author upon reasonable request.

## Abbreviations

ADC: Apparent diffusion coefficient
ASPECTS: Alberta stroke program early CT score
DWI: Diffusion-weighted imaging
DWI-R: Diffusion-weighted imaging lesion reversal
EVT: Endovascular therapy
GM: Gray matter
mRS: Modified Rankin Scale
NCCT: Non-contrast computed tomography
WM: White matter
