# Supplementary material for: Mapping DWI signal reversal and long-term tissue outcomes following endovascular therapy in acute ischemic stroke
Source: Eur Radiol. 2025 Sep 1;36(3):1721–32. doi: 10.1007/s00330-025-11943-0 (PMC12963255; doi:10.1007/s00330-025-11943-0)

Mapping DWI Signal Reversal and Long-Term Tissue Outcomes Following Endovascular Therapy in Acute Ischemic Stroke

ELECTRONIC SUPPLEMENTARY MATERIAL

Table S1. Extended Version of Tabel 1

Background characteristics and imaging features of the study cohort. This table presents data for the following subgroups: (1) The entire cohort (n=303); (2) patients included in Method 1 (n=245); (3) patients not included in Method 1 (n=58); (4) patients with diffusion-weighted imaging lesion reversal (DWI-R) >10 mm in Method 2 and follow-up imaging (n=62); and (5) patients with DWI-R lesions >10 mm in Method 2 but without follow-up imaging (n=56). P-values are provided for comparisons between subgroups 2 and 3, as well as subgroups 4 and 5.

Table S1 (Extended Version of Table 1) Demographics and imaging characteristics	Total cohort N = 303	Method 1 N=245	Missing data for Method 1 N=58	p-value Method 1 vs Missing Method 1	Follow-Up images (DWI-R>10mm) N=62	Missing Follow-Up N=56	p-value Follow-Up vs Missing Follow up
Mean age, years (SD)	68.9 (12.6)	69.2 (12.6)	67.4 (12.5)	032	66.6 (13.8)	68.4 (11.8)	0.48
Male (%)	165 (54.5)	128 (52.2)	37 (63.8)	0.15	32 (51.6)	29 (51.8)	1.0
Large vessel occlusion anterior circulation (%)	195 (64.4)	161 (65.7)	34 (58.6)	0.39	34 (54.8)	30 (53.6)	1.0
Medium vessel occlusion anterior circulation (%)	77 (25.4)	62 (25.3)	15 (25.9)	1.0	23 (37.1)	20 (35.7)	1.0
Occlusion posterior circulation (%)	31 (10.2)	22 (9.0)	9 (15.5)	0.22	5 (8.1)	6 (10.7)	0.86
Intravenous thrombolysis (%)	166 (55.0)	138 (56.6)	28 (48.3)	0.32	44 (70.9)	33 (58.9)	0.24
Median NIHSS on admission (IQR)	12 (7-17) –	12 (7-17)	13 (8-20)	0.13	11.5 (8-16)	12 (7-18)	0.86
Median time ictus to MRI pre EVT, minutes (IQR)	224 (175-312)	225 (175-306.5)	220 (190-328)	0.62	201 (145-278)	226 (176-294)	0.24
Median time MRI pre to recanalization, minutes (IQR)	95 (75-137)	93 (75-133)	111 (79-150)	0.06	84.5 (69.5-103)	84 (66-117)	0.99
Median time ictus to recanalization, minutes (IQR)	335.5 (274.75-437.5)	329 (272-432)	357 (280-451)	0.27	289 (243-385)	319 (260-397)	0.29
Median time recanalization to MRI post, hours (IQR)	21.6 (18.5-25.0)	21.8 (19.1-25.1)	21.3 (17.5-24.7)	0.12	23.0 (20.8-27.2)	20.3 (17.5-24.7)	p<0.001
Median time to follow-up MRI/CT, days (IQR)					102 (90-115)		
Median DWI-ASPECTS MRI pre EVT (IQR)	7 (6-8)	7 (6-8)	7 (6-9)	0.56	7 (5-8)	7(6-8)	0.15
Median DWI-ASPECTS MRI post EVT (IQR)	7 (5-8)	7 (5-8)	7 (5-9)	0.65	8 (6-9)	7 (6-8)	0.73
mTICI ≥2b (%)	283 (93.4)	232 (94.7)	51 (87.9)	0.12	62 (100)	55 (98.2)	0.96
mTICI ≥2c (%)	205 (67.7)	167 (68.2)	38 (65.5)	0.82	50 (80.6)	40 (71.4)	0.34
Heidelberg bleeding classification 1a&1b post EVT (%)	101 (33.3)	86 (35.1)	15 (25.9)	0.24	24 (38.7)	18 (32.1)	0.36
Heidelberg bleeding classification 1c post EVT (%)	39 (12,9)	35 (14.3)	4 (6.9)	0.20	4 (6.5)	4 (7.1)	1.0
Heidelberg bleeding classification 2 post EVT (%)	19 (6.3)	13 (5.3)	6 (10.4)	0.26	1(1.6)	0 (0)	1.0
Haemorrhage subarachnoid post EVT (%)	28 (9.2)	22 (9.0)	6 (10.4)	0.94	4 (6.5)	2 (3.6)	0.77
Pre-stroke mRS ≤2 (%)	292 (96.4)	237 (96.7)	55 (94.8)	0.76	61 (98.4)	53 (94.6)	0.54
3-month mRS ≤2 (%)	210 (70.9)	173 (72.4)	37 (64.9)	0.34	56 (90.3)	43 (79.6)	0.17

**Pre EVT MRI scanner Local Hospital (%)**	22 (7.3)	13 (5.3)	9 (15.5)	0.02	6 (9.7)	1 (1.8)	0.16
**Pre EVT MRI scanner EVT Centre 1.5 Tesla (%)**	270 (89.1)	224 (91.4)	46 (79.3)	0.02	56 (90.3)	55 (98.2)	0.16
**Pre EVT MRI scanner EVT Centre 3 Tesla (%)**	11 (3.6)	8 (3.3)	3 (5.2)	0.76	0 (0)	0 (0)	-
**Post EVT MRI scanner EVT Centre 1.5 Tesla (%)**	295 (97.4)	239 (97.6)	56 (96.7)	0.30	62 (100)	55 (98.2)	0.96
**Post EVT MRI scanner EVT Centre 3 Tesla (%)**	8 (2.6)	6 (2.4)	2 (3.4)	1.0	0 (0)	1 (1.79)	0.96

Figure S1. The video shows axial probabilistic maps in MNI space.

Panel A: Tissue regions extracted from the HarvardOxford-sub-maxprob-thr25-2mm atlas, derived from the Harvard-Oxford cortical and subcortical structural probabilistic atlases (also used as the background for panels B–F).

Panel B and C: Graphical representation of the probability of diffusion-weighted imaging lesion reversal (DWI-R). Higher probabilities are shown in darker colours. Only voxels that were part of a DWI lesion in at least five patients on MRI performed prior to endovascular therapy (EVT) are included.

Panel D: Spatial distribution of DWI lesions (blue) and DWI-R lesions (red), showing all voxels affected in at least one patient.

Panel E: Number of patients with a DWI lesion per voxel on pre-EVT MRIs.

Panel F: Number of patients with DWI-R per voxel.

Figure S2. The animation shows voxel-wise probabilities of diffusion-weighted imaging lesion reversal (DWI-R) on axial maps in MNI space. Higher probabilities are represented in darker colours.

Figure S3. The animation shows colour-coded, voxel-wise probabilities of diffusion-weighted imaging lesion reversal (DWI-R) on axial maps in MNI space.

Light yellow = less than 15% probability of DWI-R.

Dark yellow = 15–30% probability of DWI-R.

Light orange = 30–45% probability of DWI-R.

Dark orange = greater than 45% probability of DWI-R.

Figure S4. The animation shows voxel-wise probabilities of diffusion-weighted imaging lesion reversal (DWI-R) on coronal maps in MNI space. Higher probabilities are represented in darker colours.

Figure S5. The animation shows colour-coded, voxel-wise probabilities of diffusion-weighted imaging lesion reversal (DWI-R) on coronal maps in MNI space.

Light yellow = less than 15% probability of DWI-R.

Dark yellow = 15–30% probability of DWI-R.

Light orange = 30–45% probability of DWI-R.

Dark orange = greater than 45% probability of DWI-R.

Figure S6. The animation shows voxel-wise probabilities of diffusion-weighted imaging lesion reversal (DWI-R) on sagittal maps in MNI space. Higher probabilities are represented in darker colours.

Figure S7. The animation shows colour-coded, voxel-wise probabilities of diffusion-weighted imaging lesion reversal (DWI-R) on sagittal maps in MNI space.

Light yellow = less than 15% probability of DWI-R.

Dark yellow = 15–30% probability of DWI-R.

Light orange = 30–45% probability of DWI-R.

Dark orange = greater than 45% probability of DWI-R.

Figure S8. MRI images from a DWI lesion without signal changes in reversed lesion areas on follow-up imaging. Panel A shows the acute DWI image before endovascular therapy (EVT), with a large lesion indicated by a dotted circle. Panel B displays the corresponding DWI image from the day after EVT. An open arrow indicates the persistent lesion area, while a closed arrow marks a region with apparent lesion reversal. Panel C presents the corresponding FLAIR image obtained over a month later. The closed arrow indicates the region of the previously reversed lesion, showing no evidence of signal abnormalities. The open arrow indicates the area where the DWI signal persisted after EVT, with signal changes also observed on follow-up FLAIR.

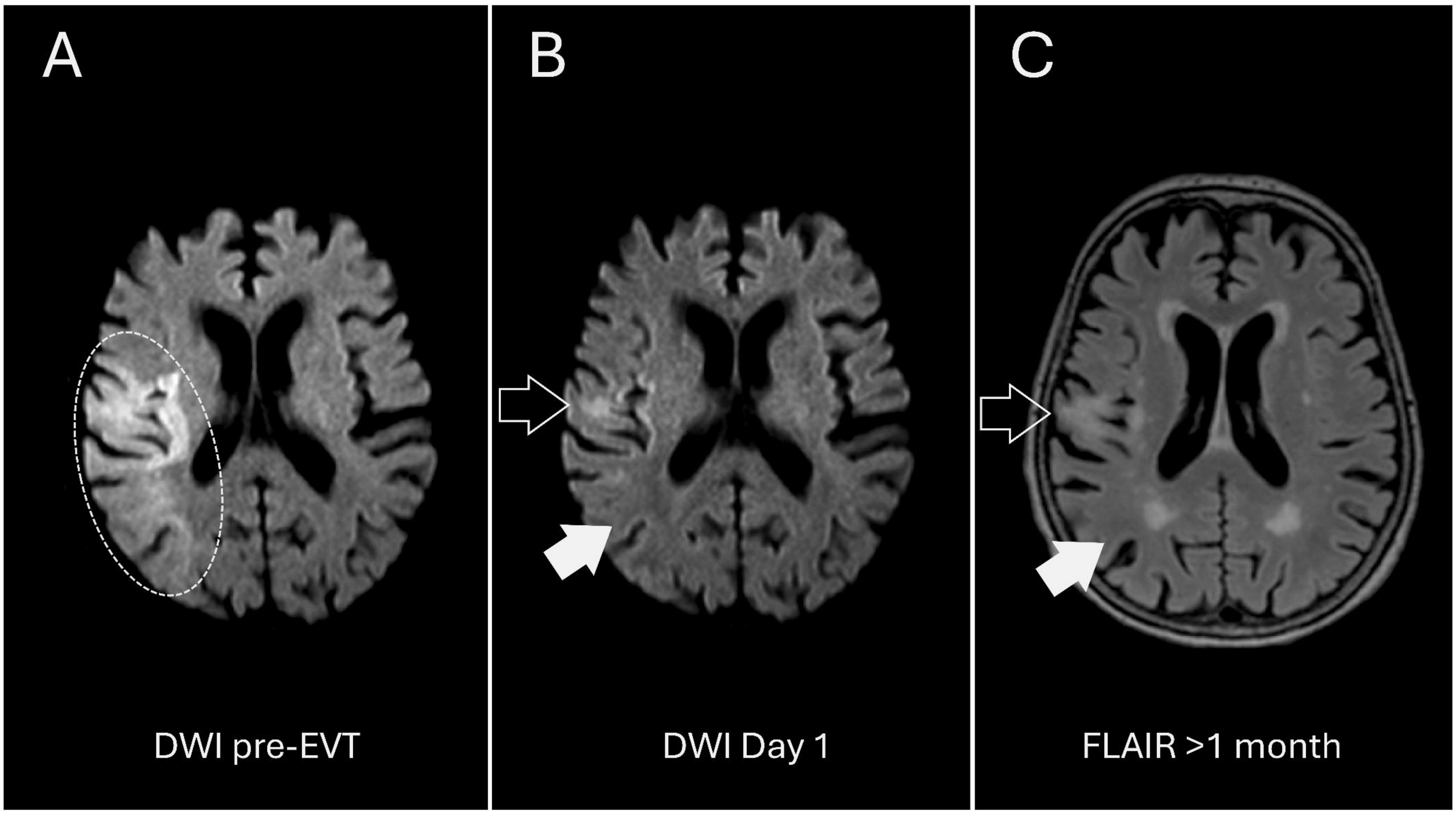

Figure S9. MRI images from a DWI lesion with subtle signal changes in initially reversed lesion areas on follow-up imaging. Panel A shows the acute DWI image before endovascular therapy (EVT), with a large lesion indicated by a dotted circle. Panel B displays the corresponding DWI image from the day after EVT. An open arrow indicates the persistent lesion area, while a closed arrow marks a region with apparent lesion reversal. Panel C presents the corresponding FLAIR image obtained over a month later, with the closed arrow indicating subtle signal alterations in the previously reversed region, suggesting minor tissue injury. The open arrow indicates the area where the DWI signal persisted after EVT, with signal changes also observed on follow-up FLAIR.

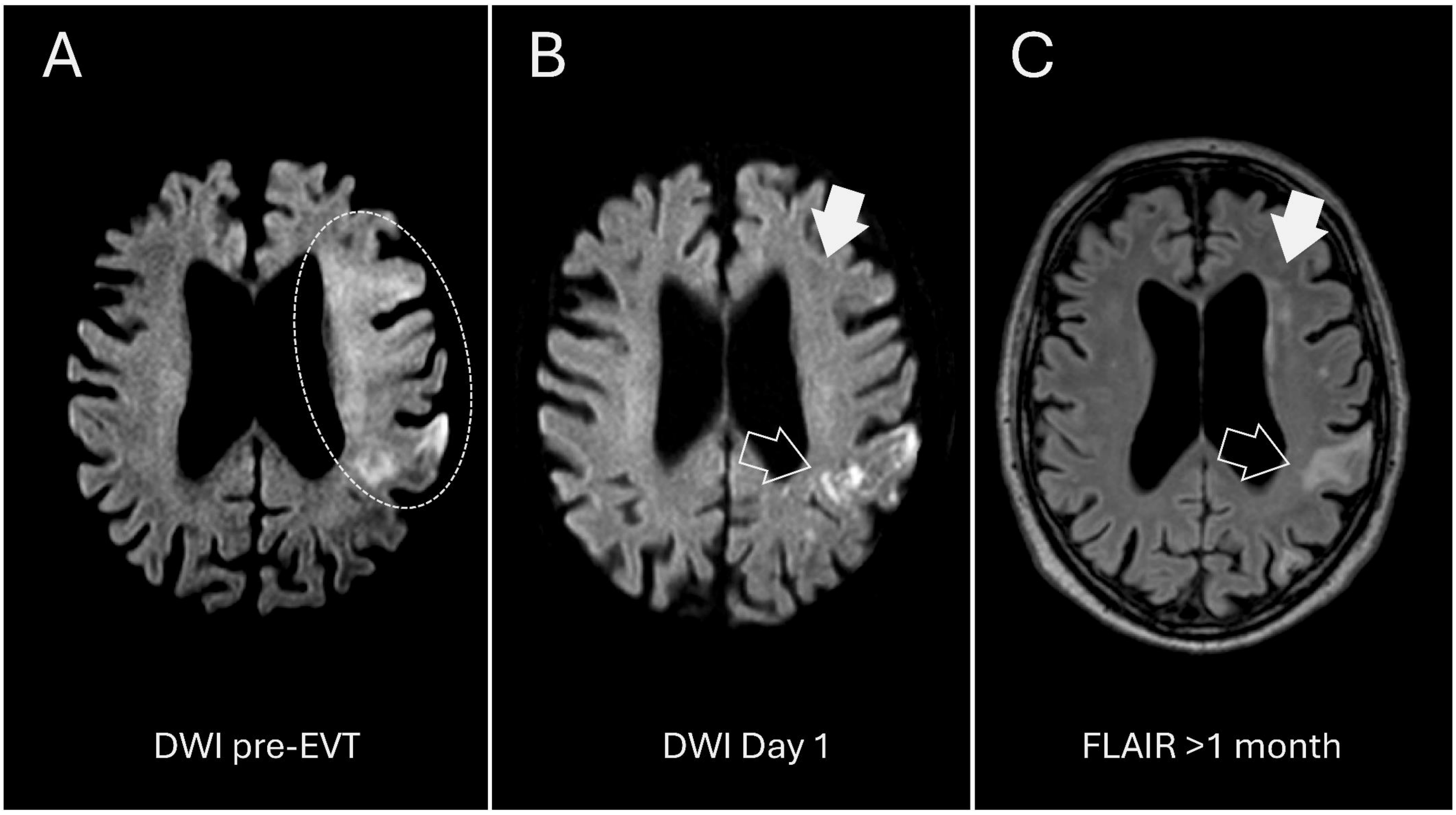

Figure S10. MRI images from a DWI lesion with severe signal changes in initially reversed lesion areas on follow-up imaging. Panel A shows the acute DWI image before endovascular therapy (EVT), with a large lesion indicated by a dotted circle. Panel B displays the corresponding DWI image from the day after EVT. An open arrow indicates the persistent lesion area, while a closed arrow marks a region with apparent lesion reversal. Panel C shows the corresponding FLAIR image obtained more than a month later. The closed arrow marks pronounced signal changes in the previously reversed region, indicating tissue damage despite early reversal. The open arrow indicates the area where the DWI signal persisted after EVT, with signal changes also observed on follow-up FLAIR.

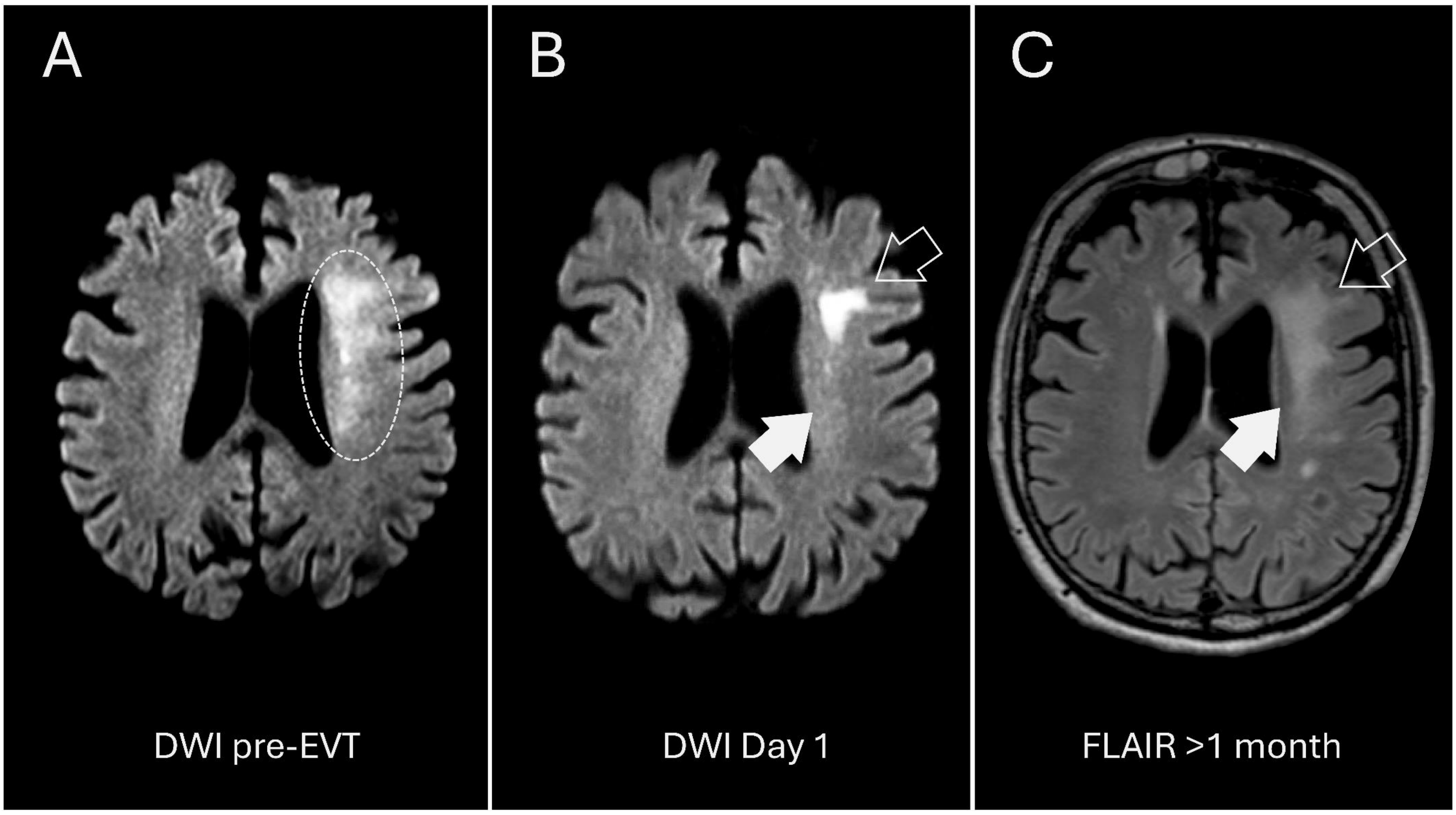

Supplement: Supplementary file 1 — ELECTRONIC SUPPLEMENTARY MATERIAL [file 330_2025_11943_MOESM1_ESM.pdf]
