# Supplementary figures and images for: Mapping DWI signal reversal and long-term tissue outcomes following endovascular therapy in acute ischemic stroke
Source: Eur Radiol. 2025 Sep 1;36(3):1721–32. doi: 10.1007/s00330-025-11943-0 (PMC12963255; doi:10.1007/s00330-025-11943-0)

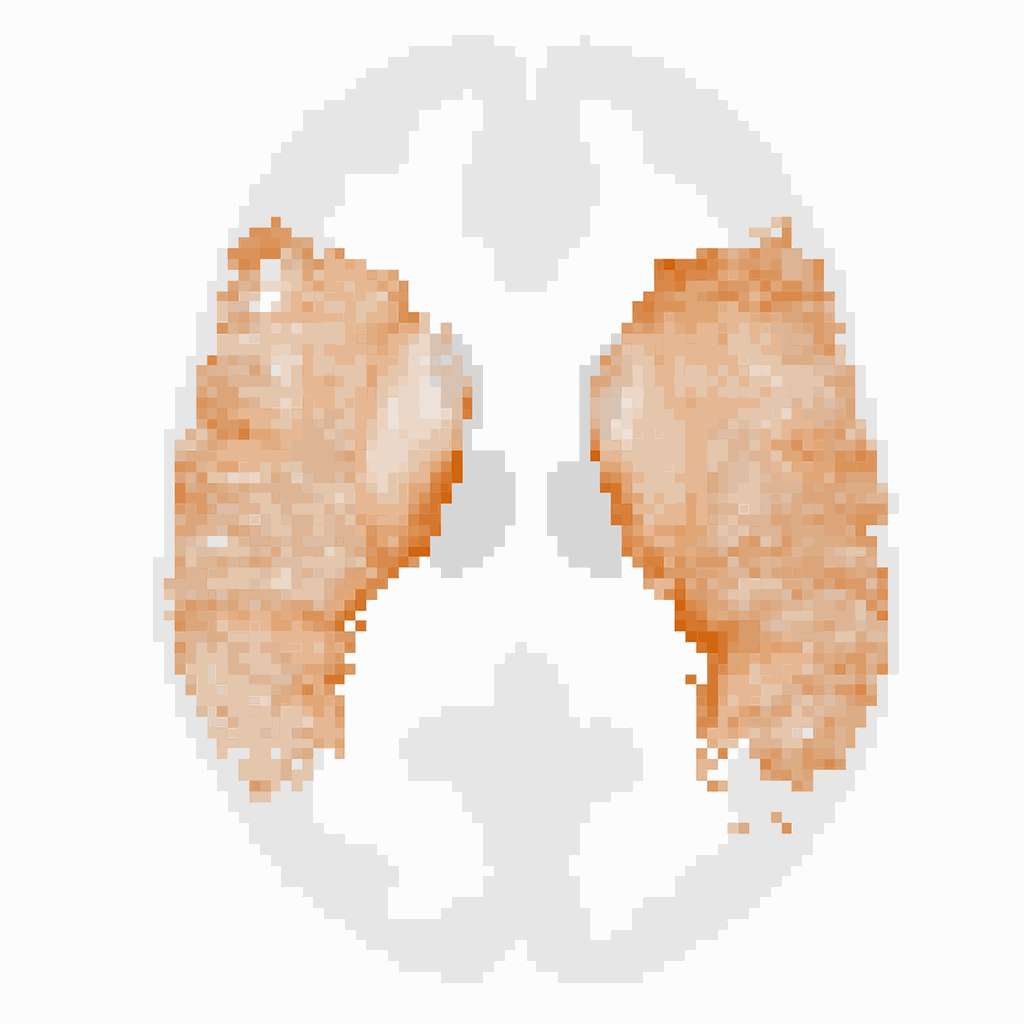

Supplement: Supplementary file 3 — Supplementary Information Figure S2 (Axial) [file 330_2025_11943_MOESM3_ESM.gif]

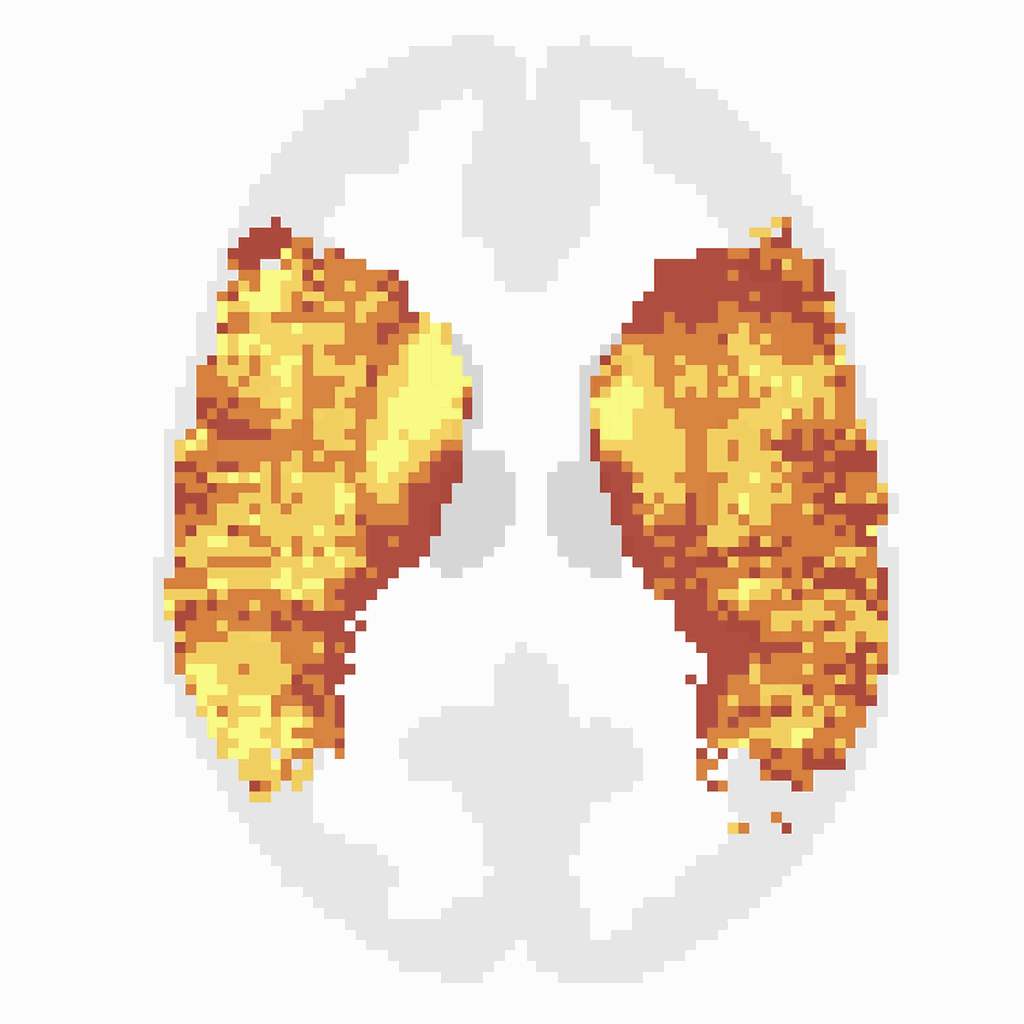

Supplement: Supplementary file 4 — Supplementary Information Figure S3 (Axial) [file 330_2025_11943_MOESM4_ESM.gif]

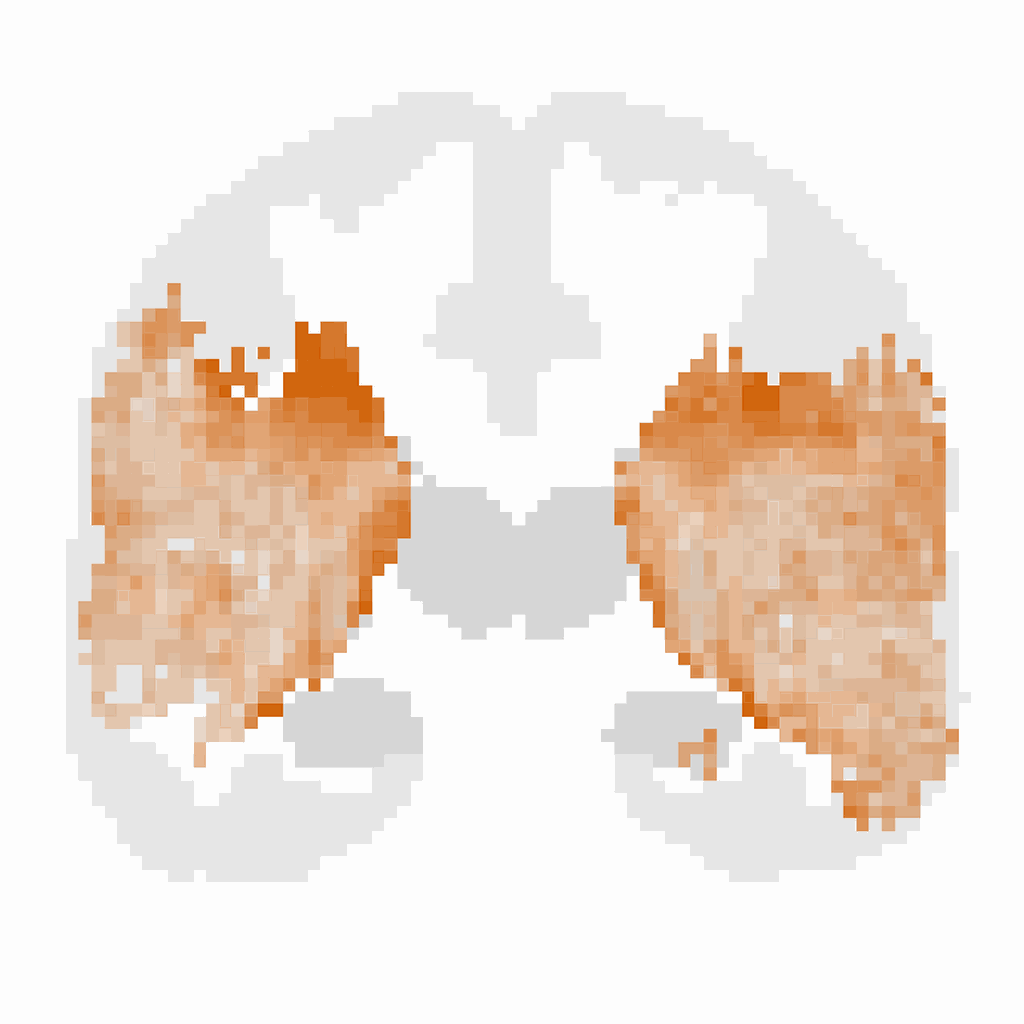

Supplement: Supplementary file 5 — Supplementary Information Figure S4 (Coronal) [file 330_2025_11943_MOESM5_ESM.gif]

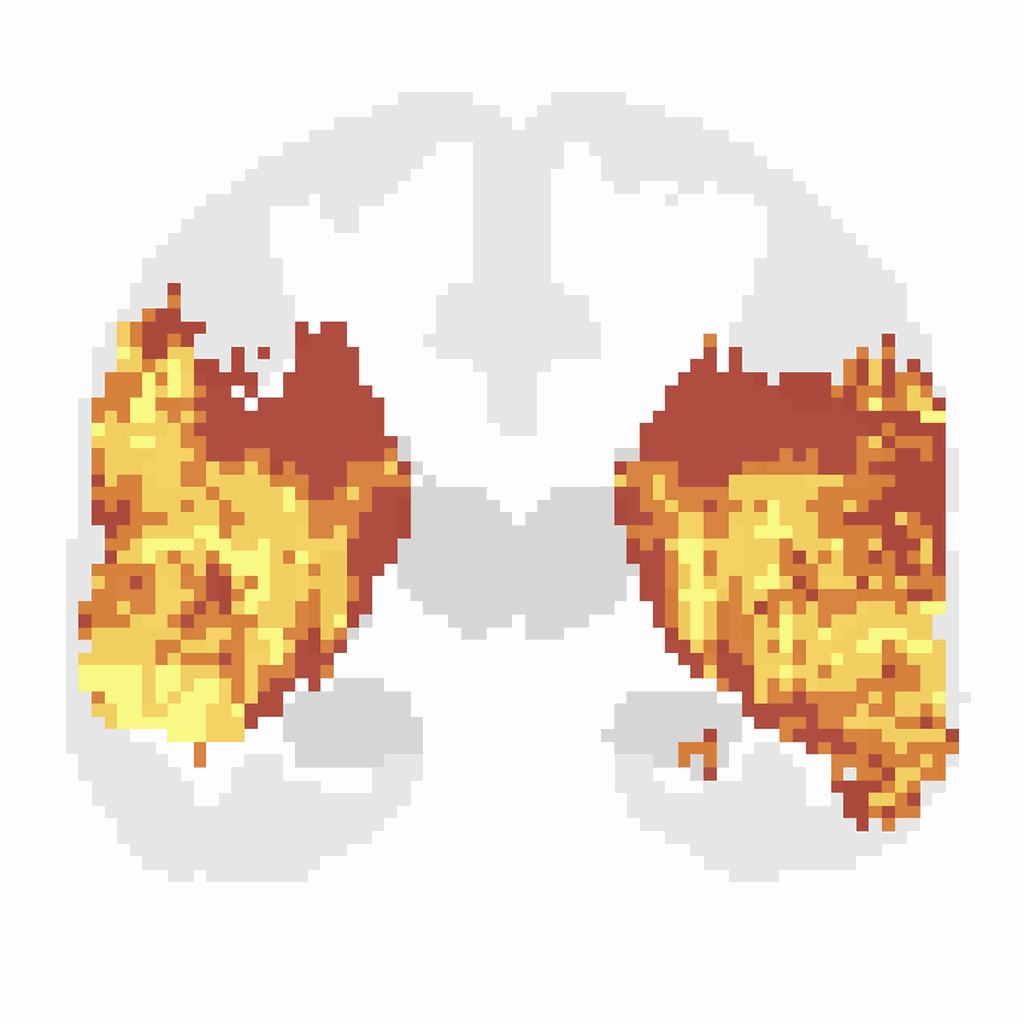

Supplement: Supplementary file 6 — Supplementary Information Figure S5 (Coronal) [file 330_2025_11943_MOESM6_ESM.gif]

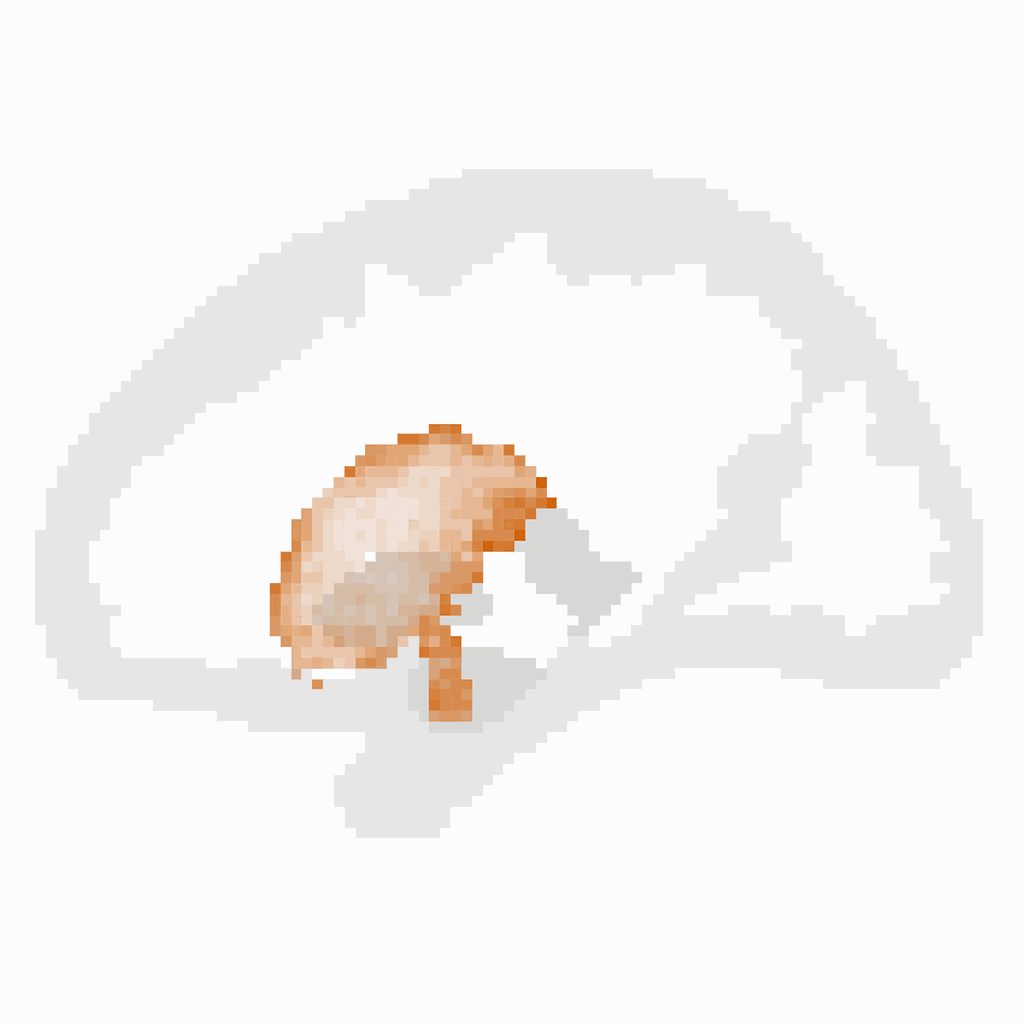

Supplement: Supplementary file 7 — Supplementary Information Figure S6 (Sagittal) [file 330_2025_11943_MOESM7_ESM.gif]

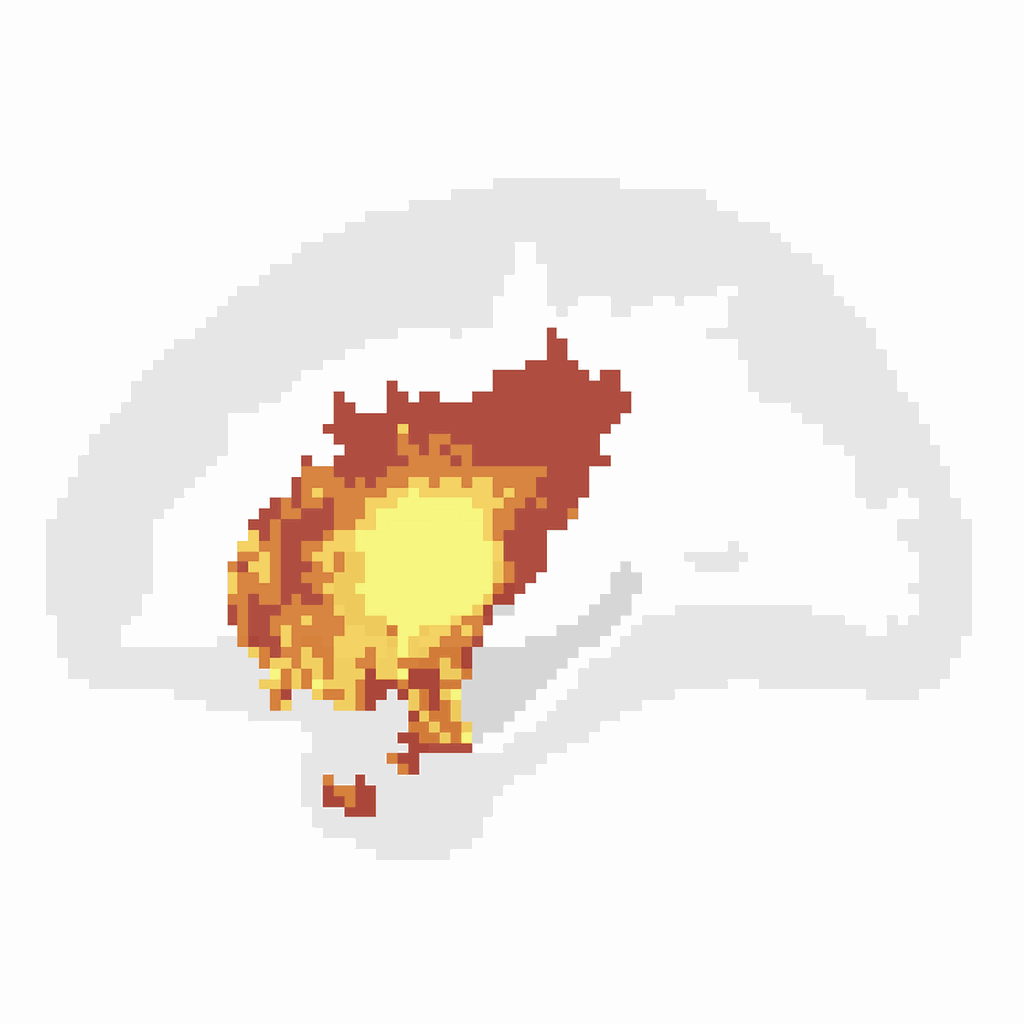

Supplement: Supplementary file 8 — Supplementary Information Figure S7 (Sagittal) [file 330_2025_11943_MOESM8_ESM.gif]
